# Recanalization of the Duodenum Following a High-Grade Post-Laparoscopic Cholecystectomy Duodenal Injury: Personalized Approach Presentation and Systematic Review of Management Options

**DOI:** 10.3390/jpm16030131

**Published:** 2026-02-28

**Authors:** Nefeli K. Tomara, Christos Doudakmanis, Dionysios Prevezanos, Ioannis Lymperis, Stylianos Kykalos, Gerasimos Tsourouflis, Chrysovalantis Vergadis, Nikolaos I. Nikiteas, Dimitrios Dimitroulis

**Affiliations:** 1 2nd Propaedeutic Department of Surgery, Laiko General Hospital of Athens, National and Kapodistrian University of Athens, Agiou Thoma 17, 11527 Athens, Greeceprevedio@hotmail.com (D.P.); tripoli13panta@gmail.com (I.L.); kykalos@gmail.com (S.K.); gtsourouflis@med.uoa.gr (G.T.); nnikit@med.uoa.gr (N.I.N.); dimdimitr@med.uoa.gr (D.D.); 2 Interventional Radiology Unit, Department of Radiology, Laiko General Hospital of Athens, Agiou Thoma 17, 11527 Athens, Greece; valvergadis@yahoo.gr

**Keywords:** duodenal injury, duodenal perforation, post-laparoscopic cholecystectomy

## Abstract

Background: Laparoscopic cholecystectomy is considered the gold standard surgical technique for the treatment of gallbladder diseases worldwide. Nonetheless, despite its safety and popularity, it comes with certain complications. Duodenal injury is an extremely rare, but potentially fatal complication. The rarity of duodenal injuries, combined with under-reporting of incidents, has resulted in a scarcity of references in the international literature. Case Presentation: We present the case of a 72-year-old male patient, initially subjected to laparoscopic cholecystectomy that was intraoperatively converted to open cholecystectomy. During the laparoscopic approach, the patient experienced a major duodenal injury, which was treated intraoperatively using primary suturing. Upon postoperative failure of the indicated surgical treatment, extended individualized salvage surgery was performed, with an ultimately favorable outcome. Methods: When assessing the overall implications of this case, we conducted a review of the published literature in English, published up to November 2025 on patients with duodenal injury after exclusively laparoscopic cholecystectomy. Results: We then categorized the 105 cases described based on the therapeutic approach: surgical, conservative, and endoscopic, with a view to attempt to classify the available therapeutic options based on the time of diagnosis, the patient’s performance status, and the size and location of the injury. Conclusions: While laparoscopic cholecystectomy is a common surgical procedure, duodenal injuries remain rare. The treatment approach highly depends on the time of injury recognition, site and extent of the injury and patient’s status. Treatment should be personalized based on the aforementioned parameters.

## 1. Introduction

Acute and chronic cholecystitis, symptomatic and in selected cases of asymptomatic gallbladder disease, in addition to other rare etiologies, is routinely addressed through laparoscopic cholecystectomy (LC). LC has become the standard operation technique worldwide since the 80s, as the safest and least intrusive technique. This led patients and insurers to expect laparoscopic treatment to deliver the best outcomes with minimal hospitalization and postoperative recovery periods.

Nonetheless, despite its safety record and popularity, LC is not without complications during and after surgery, with occasional dramatic outcomes. Bile duct injury is the most common complication during LC. Small bowel and vessel injuries are the other main complications. Among small bowel injuries, duodenal injury is an extremely rare but potentially fatal complication. Because of its rarity, case reports and case series on duodenal injuries are sparse in the medical literature. Reports mainly tend to focus on a description of individual cases and do not offer systematic analyses and treatment guidelines; thus, treatment options are personalized, and outcome predictions are very difficult. Most authors agree that the time of diagnosis is an important parameter for a favorable outcome, with early detection crucial for survival.

Herein, we describe the case of a 72-year-old male patient, initially subjected to LC that was intraoperatively converted to open cholecystectomy. During LC, the patient suffered a major duodenal injury, but due to a unique combination of the medical approach with the patient’s fibrotic response to inflammation, he managed to have a positive outcome. We present the timeline of all interventions performed in each stage of the patient’s postoperative course. We further discuss the treatment algorithm that we followed for this case of post-laparoscopic cholecystectomy duodenal injury. This rare case highlights the necessity of an individualized approach based on the type and extent of the injury and patient’s status.

## 2. Materials and Methods

The current study includes the presentation of an interesting case of post-laparoscopic cholecystectomy duodenal injury that we managed successfully in our Department. We present our approach in depth, as well as how we managed the setbacks on the patient’s hospitalization.

Subsequently, we conducted a systematic review reported in compliance with the Preferred Reporting Items for Systematic reviews and Meta-Analyses 2020 statement. Ref. [1] Two authors (N.K.T, C.D.) independently searched PubMed and Scopus Elsevier databases from inception through December 2025. Although, our systematic review was not formally registered in international databases, the research process followed appropriate techniques. The search process involved the following terms: “laparoscopic”, “cholecystectomy”, “complication”, “duodenal”, “duodenum”, “injury” and “perforation” combined with the Boolean operators AND/OR, in order to detect all studies that included patients with post-laparoscopic cholecystectomy duodenal injuries. Reference lists of the initially retrieved articles were manually screened to identify additional eligible articles. Any ensuing disagreements were resolved by a third reviewer (D.D.). Table S1 presents the PRISMA 2020 checklist.

After excluding duplicate studies, all studies that presented cases of post-laparoscopic cholecystectomy duodenal injuries in adults were deemed eligible for inclusion. We excluded studies published in non-English language or involving pediatric patients (<18 years of age). Furthermore, studies in which the duodenal injury was attributed to a surgical procedure other than laparoscopic cholecystectomy, such as conventional cholecystectomy, were also excluded.

Thirty-eight articles from 1991 to 2024 fulfilled the inclusion criteria, of which sixteen were case series, seven cohort studies, twelve case reports, one survey study and two clinical trials. The risk of bias of the included studies was calculate by two authors (N.K.T, C.D.) independently, with a summary assessment of “low”, “some concerns”, “high”, “no information”. Most of these studies were judged as having some concerns regarding risk of bias (27/39), mainly due to missing information in one or more domains. Twelve studies were assessed as low risk of bias. No study met criteria for an overall high risk based on the evaluated domains.

**Figure 1:**
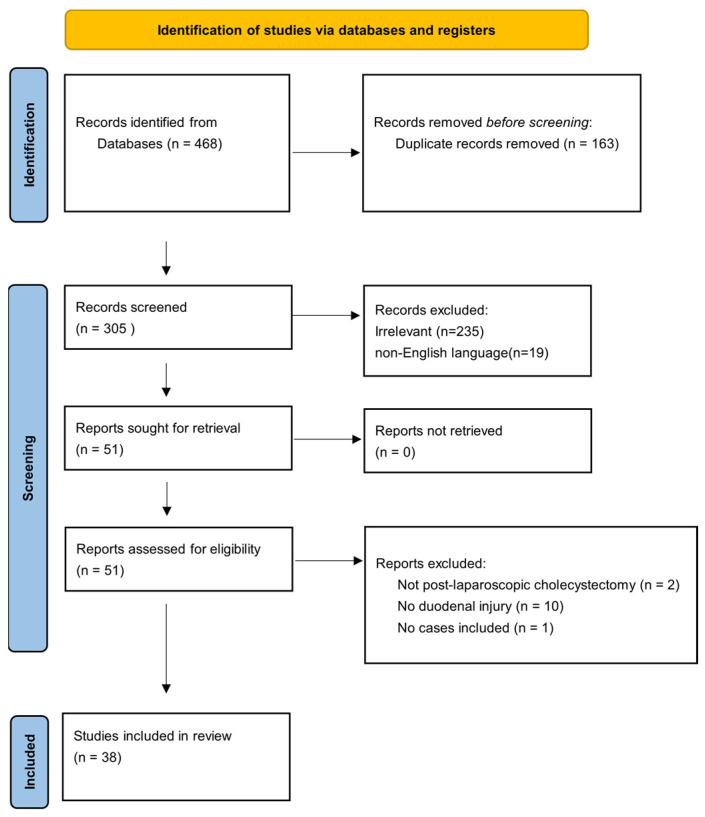
Prisma flowchart.

Data extraction was performed independently by two authors (N.K.T, C.D.) using a predefined form, which included publication details (authors, year) and type of study. Moreover, the time of duodenal injury diagnosis (intra-operative or postoperative), need of conversion to conventional cholecystectomy, as well as data concerning management approach (conservative, surgical or endoscopic) and the exact type of procedure. Finally, patient’s outcome, mortality and duration of hospital stay were also collected, were applicable (Figure 1).

## 3. Case Presentation

A 72-year-old male patient with a medical history of hypertension, depression, and obesity underwent LC for symptomatic cholelithiasis in another hospital. Due to reported marked inflammation and adhesions, the decision to convert the operation to an open procedure was made intraoperatively by the surgeon in charge, followed by laborious adhesiolysis and cholecystectomy. During the conversion to open procedure, an injury of about 1.5 cm in diameter in the descending portion of the duodenum was identified. Subsequently, based on the senior surgeon’s judgment and given the extent and location of the injury, primary repair with Graham patch omentopexy was performed, followed by a drain placement.

During the first postoperative days, the patient remained afebrile and hemodynamically stable with progressive improvement in his laboratory exams. However, on the 6th postoperative day a bilious drain outflow was observed (Table 1). Therefore, the patient was referred to our department for further treatment. Upon arrival, he was clinically distressed, pale, and febrile, with notable tachypnea and hemodynamic instability. Laboratory examination revealed elevated inflammatory markers, white blood cells (WBC) were 17.89 K/μL with neutrophilia (78.3%), c-Reactive Protein (CRP) was 127.37 mg/L, while anemia was also noted (hemoglobin 7.8 g/dL). The drain content remained bilious. Drain fluid laboratory examination revealed high bilirubin levels of 14.28 mg/dL and high amylase levels 79,538 U/L. These findings were compatible with intestinal fluids.

Further investigation with thoracic and abdominal computed tomography (CT) was deemed necessary, revealing small bilateral pleural effusions and a right lower pulmonary lobe air bronchogram image, accompanied by atelectasis. Moreover, the presence of a significant amount of right subdiaphragmatic free air with edematous postoperative elements in the hepatoduodenal space, along with the absence of any significant intra-abdominal collection, were attributed to the well-functioning drainage. After careful consideration at the given time, an emergency exploratory laparotomy during midnight was not considered an optimal option, due to the patient’s severely affected physiological reserve and the already efficient drainage. Therefore, after consultation with infectious diseases specialists, administration of empirical advanced intravenous broad-spectrum antibiotic treatment (piperacillin-tazobactam, anidulafungin) was started. Additionally, the patient received total parenteral nutrition (TPN) and intravenous fluid to compensate his daily needs and a nil per os status was initiated, aiming for the initial stabilization of patient’s condition. In order to reduce intra-abdominal exposure to bilio-pancreatic fluids, a nasogastric tube was placed as well, in combination with a continuous intravenous infusion of somatostatin (Table 1).

The next day (second day of hospitalization at referral center, and seventh postoperative day), the patient’s condition decompensated with a sudden blood drain outflow of 500 mL, leading to hemodynamic instability in need of inotropic support. Rapid resuscitation was performed with two units of packed red blood cells (RBC) and intravenous fluids, followed by an emergency CT angiography (CTA), which revealed active endoluminal extravasation of the contrast media in the descending duodenum. Elective embolization of a branch of the gastroduodenal artery was performed by an interventional radiologist, leading to clinical and laboratory responses, while content of the drain subsequently resumed to be biliopancreatic again (Table 1). Four days later (sixth day of hospitalization at referral center), another similar episode resulted in the embolization of the gastroduodenal artery itself, using coils (Table 1).

**Figure 2:**
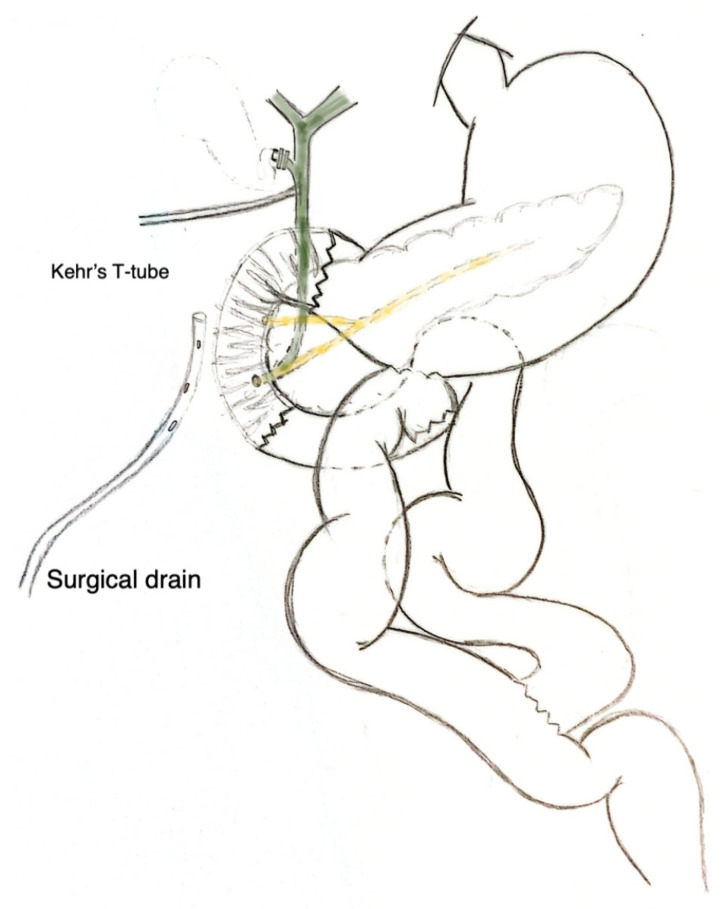
Surgical procedure.

On the eighth day of hospitalization at the referral center (14th postoperative day), the patient had a third severe episode of bleeding, which led us to the decision for an urgent surgical management attempt (Table 1). The patient underwent an exploratory laparotomy under general anesthesia, which demonstrated elimination of three quarters of the descending duodenum wall and only its posterior wall was intact (Grade IV duodenal injury). In addition, the presence of multiple blood clots both in the area of the injury, and in the omental bursa as well. Despite the thorough saline washing of the peritoneal cavity, the exact source of bleeding failed to be identified. Subsequently, a damage control procedure was carried out, based on the extent of the injury, along with the patient’s severely affected physiological reserve. The basic principles of this salvage surgical operation were the restoration of the gastrointestinal continuity, along with the adequate drainage of bilio-pancreatic fluids, as the destruction of the second part of the duodenum resulted in the release of the ampulla of Vater’s content into the peritoneal cavity. Therefore, initially ensuring patient’s survival was of paramount importance and eventually staged reconstruction in subsequent phases. This procedure included (a) pyloric exclusion and suturing the peripheral stump of the duodenum, along with a Roux-en-Y gastrojejunostomy, in order to restore the gastrointestinal continuity; (b) placement of a Kehr’s T-tube in the common bile duct to divert the bile flow; and (c) an unsuccessful attempt to catheterize the main pancreatic duct, aiming to a targeted drainage of the pancreatic fluids, resulted instead in the placement of a drain in the area. The marked inflammation of the area and the fragility of the tissues attributed to previous manipulations, the episodes of bleeding and the free-outflow of bilio-pancreatic fluids, made it impossible to clearly identify the ampulla of Vater, without creating further injuries. Therefore, given the previous complete diversion of bile fluids though the Kehr’s T-tube, and the now only outflow of pancreatic fluids from the Vater, the surgical drain placed in the area essentially acted as a targeted drainage of the pancreatic fluids. Finally, a feeding jejunostomy (FJ) was placed, aiming to restore postoperative feeding of the patient, due to his clearly affected nutritional status (Figure 2).

The absence of major intraoperative events and the patient’s stable condition throughout the procedure allowed for his immediate extubation, while he was transferred to the Intensive Care Unit (ICU), where he remained for twenty-four hours (Table 1).

Upon returning to the ward, the patient showed gradual clinical and laboratory improvement with a daily pancreatic fluid drainage of approximately 700 mL and a gradual decrease in Kehr’s T-tube outflow (a decrease from 800 mL to 100 mL per day). TPN support and enteral feeding from the FJ were well-tolerated, with restoration of gastrointestinal function and normal-colored stools on the 10th postoperative day after damage control surgery (18th day at referral center) (Table 1).

On the 12th postoperative day after damage control surgery (20th day at referral center), a bilious outflow of the surgical drain (bilirubin 40 mg/dL, amylase 172,230 U/L) was accompanied by a null Kehr’s T-tube outflow, which was attributed to Kehr’s T-tube dislocation (Table 1). Two days later, the patient became febrile (38.5 °C). In the context of fever investigation, wound suppuration was treated with drainage and debridement; additionally, based on the results of blood and drain fluid cultures, the intravenous antibiotic regimen was modified to meropenem, vancomycin and tigecycline. Furthermore, a CT scan was performed, which revealed a 5.4 × 6.7 cm intra-abdominal collection with air-fluid level and peripheral contrast enhancement at the surgical field and gallbladder bed (anatomical duodenum position), in contact with the surgical drain. Moreover, the oral contrast uptake appeared to be promoted within the collection, as in pyloric recanalization after surgical exclusion (without transaction). Based on the above, it was suspected that the Kehr’s T-tube had been moved out of the peripheral common bile duct, combined with pyloric and duodenal stump blowout, leading to the drainage of the aforementioned collection into both the peripheral portion of the duodenum and the surgical drain. Therefore, a cholangiography was performed by injecting a contrast agent via the Kehr’s T-tube, highlighting the extra-biliary location of the Kehr’s T-tube, the delayed uptake of the contrast agent into the peripheral bile duct, and then its drainage into the peripheral stump of the duodenum (Table 1).

On the 23rd postoperative day after damage control surgery, the febrile episodes relapsed, which were attributed to fungal blood infection with positive blood cultures for Candida parapsilosis, leading to remodification of intravenous antibiotic treatment to piperacillin-tazobactam and anidulafungin (Table 1). Subsequently, the patient’s condition was ameliorated, with restored gastrointestinal function along with normal-colored stools; and therefore, he was discharged on the 35th postoperative day after damage control surgery (67th postoperative day after index surgery and 61st at referral center), carrying the abdominal surgical drain, with a daily biliopancreatic fluids’ outflow of 1500 mL (Table 1).

Three months later, the patient presented to the emergency department disoriented, distressed, and pale with excessive perspiration, tachycardia, and hypotension, while reporting a daily bilious drain outflow of 1500 mL and negligible oral fluid intake. After clinical and laboratory examination, pre-renal acute renal injury was diagnosed due to dehydration which was addressed by aggressive intravenous hydration with gradual restoration of his vital signs, urination, and renal function (Table 1).

**Figure 3:**
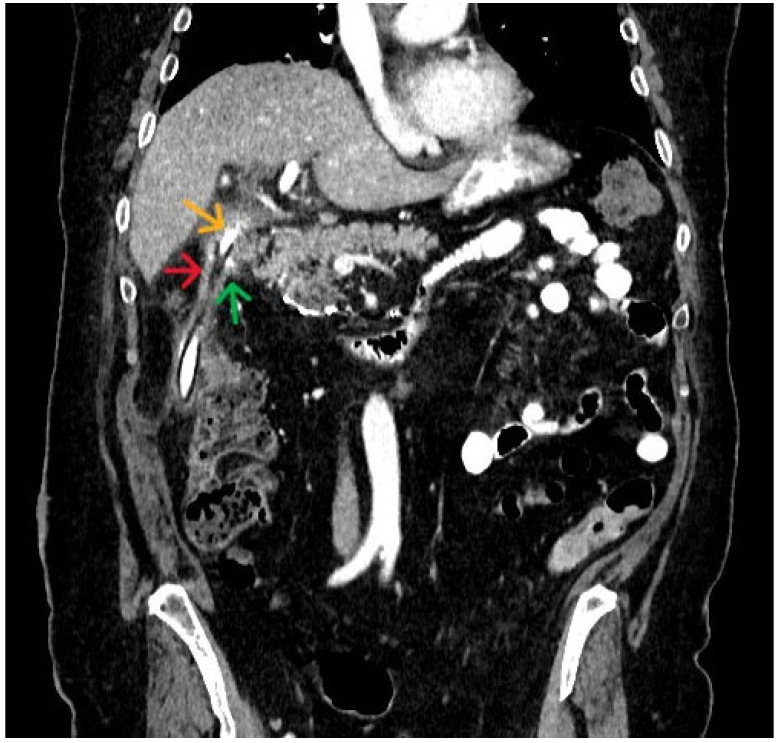
Abdominal CT scan with intravenous and oral contrast uptake demonstrating duodenum recanalization around surgical drain. Red arrow: surgical drain, Yellow arrow: recanalization of central duodenum, Green arrow: recanalization of peripheral duodenum.

As part of the patient‘s re-evaluation, a CT scan was performed without detecting any intra-abdominal collection; however, in the anatomical position of the duodenum, a newly formed fistulous tubular formation was observed, with the end of the surgical drain tangent to its wall. After consideration, creation of this fistulous tract was attributed to the interaction of fluid flow passing through the postoperative fibrous elements of the area. This fibrosis is the result of chronic inflammation caused by both surgical intervention and pancreatobiliary fluids outflow. Moreover, after administering the oral contrast agent, we observed its complete passage into the peripheral duodenal stump without any suspicion of contrast escape into the gastrohepatic space. In addition, after the administration of a contrast agent through the surgical drain, its complete advancement to the peripheral duodenum was revealed. Thus, a matured fistulous tract connecting both proximal and distal duodenal stumps was created, using the fibrous tissue around the surgical drain as newly formatted wall (Figure 3).

**Figure 4:**
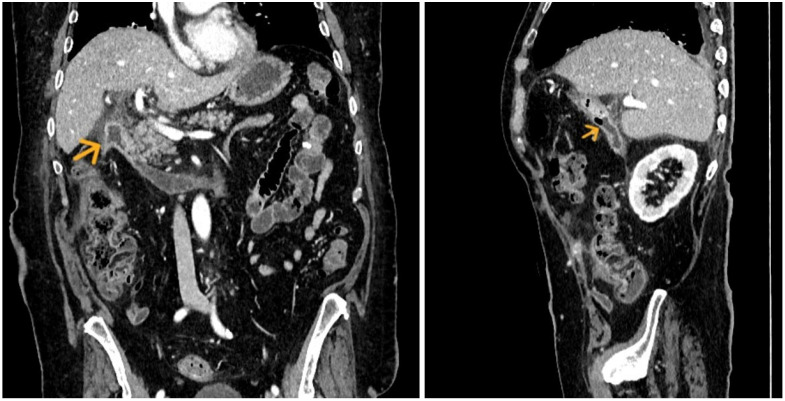
Abdominal CT scan with intravenous contrast uptake demonstrating duodenum recanalization after surgical drain’s removal. Yellow arrow: duodenal recanalization.

This led to the decision to remove the drain (Table 1, Figure 4).

Further endoscopic examination was not deemed necessary, as the patient remained asymptomatic and in order to avoid an iatrogenic complication in an attempt to satisfy medical curiosity. The following day, an ultrasound was performed with absence of any abdominal collection, and the patient was discharged.

At the annual follow-up, after quarterly reassessments, the patient appeared to be “reborn” (Table 1). He was fully mobilized and independent, with a complete reintegration into his daily routine. An undisturbed gastrointestinal function was reported, along with an excellent nutritional status, while his laboratory test results were unremarkable.

## 4. Discussion

Laparoscopic cholecystectomy (LC) was introduced in the 1980s, with the first LC performed by Prof. Dr. Eric Muhe in Boblingen, Germany, on 12 September 1985 [1]. Initial disbelief, apprehension, and criticism were followed by many randomized studies and meta-analyses comparing the new technique’s intraoperative and postoperative complications and patients’ recovery time, to those achieved under the open cholecystectomy technique [2,3]. The new technique demonstrated notable advantages, such as reduced morbidity and mortality rates, as well as reduced postoperative hospital stay and recovery periods. Additional improvements in postoperative pain were also observed, without any increase in operative time and severe hemorrhage rates [4,5]. The clear patient safety advantages of the new technique were combined with clearly enhanced cost-effectiveness, a major issue for all healthcare systems [6]. In view of these outcomes, it is not surprising that within the next two decades, LC has become the gold standard surgical technique worldwide for the treatment of gallbladder diseases and, at the same time, has played a pivotal role in the development and consolidation of Minimally Invasive Surgery.

LC is one of the most performed abdominal surgical techniques, with approximately 750,000 performed annually in the United States [7]. Complications following LCs may occur in up to 2% of patients undergoing surgery [8], which is of major importance considering the number of operations performed. With the main aim of avoiding and, as far as possible, preventing potentially devastating complications, the pioneer surgeon Steven Strasberg formulated the concept of “Critical View of Safety,” which is recognized and adopted by the global surgical community as the most fundamental operative principle when performing LCs [9,10].

Bile duct injury is the most common complication encountered after LC, with an overall risk of 0.3–1.8% [11]. On the other hand, extra-biliary injuries, consisting of vascular and intestinal injuries, are considered more serious due to increased morbidity and mortality rates. Intestinal injuries include those to the stomach, duodenum, small intestine, and colon. The overall incidence of duodenal injuries after LC is rather low [8], with a reported frequency of approximately 0.04% [12], but they are the most common amongst intestinal injuries [13]. The literature suggests that the most affected part of the duodenum is the second part, followed by the first and the third parts [8]. However, a recent study indicated that the first part of the duodenum was the most frequently affected in their patients [12].

Duodenal injuries are mostly the result of thermal injury caused using electrocautery during dissection, but also by retraction and blunt or sharp dissection [8]. Most of such injuries are spotted intraoperatively, which leads to their immediate treatment, and therefore, to optimal patient outcomes with lower related morbidity and mortality rates. However, detection of such injuries may also take place several days after surgery, potentially leading to severe implications for the patient, such as peritonitis and sepsis [14]. Delayed detection of duodenal injuries also results in increased mortality, with the literature reporting a rate of up to 18% [8]. Reviewing the literature for patients with duodenal injuries after exclusively undergoing LC (105 patients), the time of diagnosis was reported in 63 patients (60%), while there was no record for the remaining 42 (40%) [13,15,16,17,18,19,20,21,22] (Table 2 and Table 3).

Of these 63 patients, intraoperative detection was possible in only 29 patients (46%) [12,23,24,25,26,27,28,29,30,31,32,33,34,35]. Moreover, from the postoperative detection group, early postoperative diagnosis (<48 h from the operation) occurred in 15 patients (23.8%) [12,16,17,18,19,23,36,37,38,39,40,41] and late (>48 h) in 17 (27%) [12,17,23,30,36,42,43,44,45,46,47,48,49]. For the remaining two patients (3.2%) the exact time of postoperative diagnosis was not clearly determined [50,51] (Table 3). Overall mean time to diagnosis, and thus further treatment, was 3.4 days. However, if we were to remove a single patient who remained undiagnosed until three months postoperatively [47], the mean time to diagnosis was then reduced to 1.9 days (Table 2). Additionally, the literature review indicated a mortality rate of 11.45%, which is lower than the one indicated in the most recent published study [12] (Table 2 and Table 3).

**Table 2:** Literature review of post-laparoscopic cholecystectomy duodenal injuries.

Reference	Number of Patients	Time of Diagnosis (Days)	Conversion	Management	Specified Procedure	Outcome	Hospital Stay
*Jing et al.* [46]	1/3000	4		C	percutaneous drainage	alive	26
*Modi et al.* [41]	1	2		C	existing surgical drain	alive	24
*Taylor et al.* [37]	1/170	1		S	PR	alive	26
*Berry et al.* [44]	1	6		S	pyloric exclusion + GJ + T-tube duodenostomy + FD	alive	>20
*Eden et al.* [45]	1	12		S	omental patch	alive	NA
*Testini et al.* [23]	5	5		S (x2)	**1st:** Petzer duodenostomy **2nd:** gastric resection + duodenal stump closure	died	90
		3		S	Roux-en-Y duodenojejunostomy	alive	45
		INTRA-OP	NA	S	PR over T-tube	alive	11
		1		S	PR over T-tube	alive	15
		4		S	duodenopancreatectomy	alive	62
*Gaillard et al.* [31]	1	INTRA-OP	No	E	stent + NJF	alive	>72
*Gupta et al.* [15]	1/42	NA	NA	S	PR + T-tube duodenostomy + FJ	alive	NA
*Jakhmola et al.* [40]	1	2		S + C + S	**1st:** T-tube duodenostomy + FJ **2nd:** (2 injuries) PR larger, T-tube duodenostomy over smaller one + pyloric exclusion + FJ	alive	120
*Angelopoulos et al.* [36]	2	1		S	PR and omental patch	alive	11
		8		S (no diagnosis) + C (after diagnosis)	lap washout + surgical drainage	alive	20
*Croce et al.* [16]	4/2100	1		S	PR	alive	NA
		NA		S x 2 (no diagnosis) + C (after diagnosis)	**1st:** surgical drainage **2nd:** retroperitoneal surgical drainage	alive	60
		2		S	PR and omental patch	alive	NA
		NA		S	PR	alive	NA
*Greenbaum et al.* [42]	1	3		S (x2) + C + E	**1st:** PR and omental patch Multiple surgical washouts + drainage **2nd:** duodenostomy tube + biliary diversion C: percutaneous FJ +PTBD E: esophageal stent	alive	NA
*Kwon et al.* [24]	2/1190	INTRA-OP	No	S	PR (endo-GIA)	alive	11
		INTRA-OP	No	S	PR	alive	10
*El-Banna et al.* [30]	4	4		C	percutaneous drainage	died	NA
		3		S	gastrectomy + duodenostomy	alive	NA
		4		S	gastrectomy + duodenostomy	died	NA
		INTRA-OP	NA	S	serosal patch	died	NA
*Ward et al.* [19]	2/29	NA		S	PR	alive	NA
		2		S	PR	died	NA
*Ress et al.* [17]	3/22	4		S	PR and omental patch	died	14
		NA		S	PR	NA	NA
		1		NA	NA	NA	15
*Isaguirre et al.* [49]	1	8		E	vicryl meshplug fixed with endoclips + NJT	alive	NA
*Farooq et al.* [35]	1/247	INTRA-OP	Yes	S	NA	NA	NA
*Shakeel et al.* [47]	1	90		C	percutaneous drainage	alive	NA
*Roviaro et al.* [48]	1/1007	12		S	PR	alive	13
*Marakis et al.* [50]	1/1225	POS-OP		S	PR	alive	25
*Avrutis et al.* [43]	1	9		S	distal gastrectomy + GJ + duodenal stump closure over T-tube + FJ	alive	NA
*Bishoff et al.* [20]	1/915	NA		S	PR	NA	NA
*Schrenk et al.* [25]	2/1690	INTRA-OP	Yes	S	PR	alive	NA
		INTRA-OP	Yes	S	PR	alive	NA
*Yajima et al.* [32]	1/407	INTRA-OP	Yes	S	NA	NA	15
*Yamashita et al.* [26]	1/1054	INTRA-OP	Yes	S	PR	alive	NA
*Kum et al.* [27]	1/49	INTRA-OP	No	S	PR	alive	10
*Jena et al.* [38]	1	1		S	PR	died	NA
*Cala et al.* [39]	1/1000	2		S	PR	alive	NA
*Singh et al.* [28]	3/1748	INTRA-OP	Yes	S	PR	alive	NA
		INTRA-OP	Yes	S	PR	alive	NA
	f	INTRA-OP	Yes	S	PR	alive	NA
*Haque et al.* [29]	1/1420	INTRA-OP	No	S	PR	alive	NA
*Chen et al.* [33]	1/2428	INTRA-OP	Yes	S	NA	NA	NA
*Baev et al.* [51]	1/700	POST-OP		S	NA	NA	NA
*Huang et al.* [13]	19/39,238	NA	NA	NA	NA	NA	NA
*Wherry et al.* [21]	4/9054	NA	NA	NA	NA	1 died 3 alive	NA
*Deziel et al.* [22]	12/77,604	NA	NA	S	NA	1 died	32
*Peters et al.* [18]	2/283	2		S (x2)	**1st:** PR **2nd:** NA	died	NA
		NA	NA	S	PR over T-tube	alive	NA
*Malik et al.* [34]	5/1046	INTRA-OP	Yes (5)	S	NA	NA	NA
*Diaz-Martinez et al.* [12]	11/15	INTRA-OP	No	S	PR	alive	NA
		INTRA-OP	No	S	PR + FJ	alive	NA
		INTRA-OP	No	S	PR	alive	NA
		INTRA-OP	Yes	S	PR	alive	NA
		6		S	PR	died	NA
		6 h		S	PR	alive	NA
		2		S	PR	alive	NA
		2		S	PR	alive	NA
		INTRA-OP	Yes	S	PR + GJ	alive	NA
		INTRA-OP	Yes	S	PR	alive	NA
		INTRA-OP	No	S	PR	alive	NA
***Our Patient***	1	INTRA-OP	Yes	S + C + S	**1st:** PR + omental patch **2nd:** pyloric exclusion + Roux-en-Y gastrojejunostomy + biliary diversion + surgical drainage of pancreatic fluids + peripheral duodenal stump closure + FJ	alive	55

NA: Not available, INTRA-OP: Intraoperative, POST-OP: Postoperative, C: Conservative, E: Endoscopic, S: Surgery, PR: Primary repair, GJ: Gastrojejunostomy, FJ: Feeding jejunostomy, NJT: NasoJejunal feeding tube, PTBD: Percutaneous transhepatic biliary drainage.

**Table 3:** Literature review of post-laparoscopic cholecystectomy duodenal injuries: time of diagnosis and mortality rates.

	N^o^ of Patients	Percentage (%)
**Time of Diagnosis**	63/105 (available data)	60
**Intra-op**	29	46
**Post-op** *Early (<48 h)* *Late (>48 h)* *N/A*	34 15 17 2	54 44.1 50 5.9
**Data availability regarding outcomes**	74/105	70.47
**Overall Mortality**	11	14.86
• **Intra-op recognition**	1	9.1
• **Post-op recognition** *Early (<48 h)* *Late (>48 h)*	8 3 5	72.7 37.5 62.5
**NA**	2	18.2

Irrespective of the exact time of injury diagnosis (i.e., intra- or postoperative), timely reparatory surgical intervention plays a crucial role in addressing the injury and improving patient prognosis [15]. Indeed, the further away we move from the intraoperative injury recognition, a progressive decrease in survival rate is observed, with survival rates reaching 94% on the first postoperative day and then dropping to 80% on the second day [12]. Available management options can be divided in three main categories. Surgical treatment options are more widely used and could also be consider as the gold standard approach. Alternatively, conservative treatment is also mentioned in the literature, as well as endoscopic treatment, which has, at least for now, a small place in management approaches with sporadic cases reported but promising results.

Several surgical techniques are used to repair duodenal injuries. The choice of the appropriate technique should be based on the time of duodenal leakage diagnosis, the site of the defect in relation to the ampulla, and the patient’s performance status. Injuries recognized intraoperatively or in the early postoperative period could be treated with primary suturing, with or without an omental patch, laparoscopically, or after conversion, depending on surgeon preference and experience [16,17,23,36,42]. Primary suturing can be reinforced using a serosal patch of the antimesenteric border of a jejunal loop [52]. Alternatively, the restoration can be performed with primary repair over a T-tube [18,23]. The literature review indicates that primary suturing constitutes the main technique when repairing injuries spotted intraoperatively or within hours after LC (31 out of 42 patients), with a mean time to diagnosis in these patients being 0.55 days [12,16,18,19,23,24,25,26,27,28,29,36,37,38,39] (Table 2 and Table 4). Successful results are described in the majority of cases, with only five reported deaths. In two of these cases, injury was identified after the second postoperative day, while in the other three cases with the injury was recognized on the first and second postoperative days [12,17,18,19,38] (Table 2 and Table 3).

Restoration of duodenal injury with the primary repair technique is not feasible in cases of delayed treatment due to the ensuing regional peritonitis and erosion of the duodenal wall. In fact, in cases of intraoperatively unrecognized injury, and thus in the absence of immediate adequate treatment, the late onset leakage could be recognized as part of the signs of sepsis and peritonitis, as well as through the presence of bilious or pancreatic contents from the abdominal drainage. In these patients, the mean time to diagnosis, based on the literature review, is 10.9 days, as shown in Table 2. When faced with this type of iatrogenic injury, initial “watch and wait” strategies are often unsuccessful, thus requiring surgical intervention. Gastric resection with gastrojejunostomy is an adequate intervention for injuries above the ampulla [23,30,43]. Other surgical approaches include duodenal drainage with feeding jejunostomy [23,40] and pyloric exclusion accompanied with gastrojejunostomy and T-tube duodenostomy [40,44]. Pyloric exclusion performed using a gastrointestinal stapler requires a gastrojejunostomy, while in those performed with sutures through gastrotomy, pylorus patency tends to be restored after a few weeks [52]. Restoration with an omental patch alone has also been used to treat delayed duodenal injury, due to the inability of primary suturing in the context of regional peritonitis and erosion of the duodenal wall [45] (Table 2 and Table 4).

In our patient, LC was quite challenging due to marked inflammation and adhesions, along with a background of morbid obesity. Due to difficulties in dissection and identification of the hepatocystic triangle, and therefore, the inability to achieve “Critical View of Safety,” the decision was made to convert the LC to an open procedure. Duodenal injury to the anterior wall of the descending part without affecting the ampulla was first noticed intraoperatively during the open procedure and was attributed to the laparoscopic part of the operation. Immediate primary repair with Graham patch omentopexy was performed, and a surgical drain was left in place. However, the defect relapsed on the sixth postoperative day with bilious drain outflow.

Larger defects of the duodenal wall may require extensive repairs. In these cases, a Roux-en-Y duodenojejunostomy or even a pancreaticoduodenectomy may be needed [23]. Symeonidis et al. presented a case series of duodenal defects repaired using a Roux-en-Y duodenojejunostomy [53]. While this procedure was performed under suboptimal conditions, as these patients are often unstable and the local conditions are not appropriate for anastomosis, the results were quite promising. All patients started enteral nutrition within the first postoperative week, with no signs of leakage.

Endoscopic treatment of duodenal injuries after LC can be considered an alternative treatment option under certain circumstances. To achieve desirable results, the absence of uncontrolled sepsis is necessary. However, in the majority of cases, a combined treatment will be needed with a simultaneous percutaneous drain placement under imaging guidance (ultrasound or mainly computed tomography) in order to manage intra-abdominal collections. Three cases of endoscopic treatment have been described in the literature: two as initial treatment immediately after the recognition of the injury at the duodenal bulb, and the third as an alternative management option after failure of the initial surgical treatment. The first such case, reported by Gaillard et al., described the placement of a coated metal stent prosthesis that migrated and needed a second endoscopy to be repositioned and fixed in place with hemostatic clips, with the combination of two double pigtail drains across the duodenal defect to decrease the fistulous output and a nasojejunal feeding tube placement passing through the stent to ensure enteral nutrition [31]. In the second study, Isaguirre et al. described the placement of a vicryl mesh plug fixed with endoclips, in combination with a nasojejunal feeding tube and nasogastric tube, leaving in place the already existing subhepatic drain [49]. Both studies described positive patient outcomes without the need for further intervention. The third case concerns a fully covered esophageal stent placement to treat duodenal injury after failure of the initial surgical treatment [42] (Table 2 and Table 4).

While endoscopic treatment of duodenal injuries constitutes an alternative method to the commonly used surgical treatment, it is still at a very early stage of adoption, especially when compared to the well-established endoscopic treatment of injuries and postoperative complications of the esophagus. This is mainly due to the difficulty of sealing the duodenum compared to the sealing of the esophagus, because of the cumulative flow of bile, pancreatic, and more easily manageable stomach fluids, as well as the frequent but treatable migration of implanted stents.

Another possibility would be to follow a conservative management, that requires complete control of sepsis, presupposes the absence of peritonitis, and a satisfactory drainage of possible intra-abdominal effusions, either with the already existing surgical drains, or by placing new percutaneous drains using imaging guidance. It is therefore addressed to patients to whom duodenal injury is identified immediately postoperatively, possibly due to bilious drain outflow, maintaining an excellent performance status with absence of peritonitis and sepsis. However, this group of patients will mostly benefit from immediate surgical treatment, usually with primary repair, if possible. Modi et al. reported conservative management of a duodenal injury after LC identified on the second postoperative day using a bilious drain, which was further used as a tube duodenostomy [41]. Therefore, the second group of patients addressed are those with delayed recognition of duodenal injury. Jing et al. [46] reported conservative management in a patient with duodenal injury identified on the 4th postoperative day in the context of fever investigation, with the emergence of a hepatic portal effusion. Percutaneous drainage, withdrawal of oral feeding, and nutritional support followed, without signs of peritonitis and the resolution of fever, but with a background of Billroth II subtotal gastrectomy and gastrojejunostomy. Additionally, El-Bana et al. reported conservative management with percutaneous drainage after late injury identification on the fourth postoperative day in the context of septic shock with a fatal outcome [30]. Shakeel et al. reported a 3-month post-LC undiagnosed duodenal injury with a continuous daily bilious outflow from the percutaneous drainage, with a positive outcome [47]. Angelopoulos et al. and Croce et al. reported one patient each with duodenal injury after LC, who underwent abdominal cavity washout and drainage without recognition of the injury, which occurred later during imaging studies [16,36]. Based on the progressive improvement in both patients, no further intervention was required. Conservative management is a time-consuming process that requires long-term hospitalization (that could vary from 24 days, 26 days, 3 months, 20 days in different studies), during which it is necessary to ensure the nutrition of the patient, as well as to continuously evaluate the patient response to treatment and the possible need for further intervention [36,41,46,47].

Therefore, this is a case of failure of the otherwise appropriate and recommended surgical treatment with primary repair and omental patch procedure for the intraoperative identification of duodenal injury after LC [54]. In addition, the subsequent conservative management that was followed, with maintenance of the surgical drain used as a T-tube duodenostomy, administration of intravenous antibiotics, withdrawal of oral feeding, placement of a nasogastric tube, and ensuring nutrition with total parenteral nutrition, control of intra-abdominal effusions, and sepsis with continuous close monitoring, was unsuccessful, resulting in a hemodynamically unstable septic patient with peritonitis.

As observed in the subsequent exploratory laparotomy, more than 75% of the circumference in the second part of the duodenum was eliminated. Therefore, according to the AAST organ injury scale for duodenal injuries, it was classified as a Grade IV injury, which is included in the Severe WSES class III duodeno-pancreatic and extrahepatic biliary three injuries [54]. Nevertheless, due to the patient’s coexisting hemodynamic instability, the injury was upgraded to a severe WSES class IV [54]. Besides the initial injury and potential erosion from the bile and pancreatic fluids, this dehiscence could also be attributed to ischemic alterations following the two transarterial embolizations.

Transarterial embolizations may have attributed to the ischemic alterations of the duodenum. In the literature, it is reported that ischemic complications following GDA embolization could be present in 7 to 16% of the patients [55,56]. Besides duodenal wall ischemia, these ischemic events also include duodenal stenosis and ischemic pancreatitis [55]. The cases of transmural duodenal ischemia still remain low [56]. There are several risk factors associated with ischemia following GDA embolization. These factors include prior abdominal surgery, hemodynamic status, and the technique of the embolization. This being said, placement of the embolics distally rather than proximally could result into higher incidence of ischemia. One should take into consideration the alterations in the blood supply. Duodenum has dual blood supply through the celiac axis and superior mesentery artery. There is evidence that in a significant percentage of patients undergoing GDA embolization, there is celiac axis stenosis which could alter the overall perfusion [56,57].

Pseudoaneurysms of the GDA are the result of erosion by inflammation. This could be iatrogenic, attributed to postoperative inflammation, but it also could be a result of pancreatitis. In these cases, duodenum wall is compromised by the chronic inflammation and its ischemia could not be attributed solely on GDA embolization [58,59]. Ischemia following embolization for blunt trauma is extremely rare. Patients affected are mostly younger patients and the transarterial embolization takes place in a superelective manner, and thus there is a lower incidence of ischemia [59].

Our main concern at that critical point was to restore gastrointestinal tract continuity to enable the patient to receive enteral feeding and adequate drainage. The intraoperative findings did not provide evidence that duodenojejunostomy was feasible due to the extent of the defect, while the patient was already unstable with poor performance status; thus, it was a poor candidate for an extensive operation, such as a pancreaticoduodenectomy. Therefore, according to the 2019 WSES-AAST guidelines, a damage-control strategy with possible staged reconstruction in subsequent phases was the alternative optimal approach [54]. While, at some point our approach seemed to fail, with an initially undesirable opening of the central and peripheral duodenal stump, finally the formation of fibrous tissue that was developed as a response to the inflammation restored the patency of the duodenum, in a manner of tight “neoduodenum” formation.

There are certain limitations in our study. Post-laparoscopic cholecystectomy duodenal injuries are extremely rare, but also they are underreported. The existing literature consists of either case reports or small case series. The heterogeneity in the reported data was also noted. This literature had been published in a wide time span. In this manner, assessment of older reports as well was deemed necessary. In the literature, there is heterogeneity in the type of the repair proposed. The decision of the type of the repair is based on the time of the diagnosis, the extent of the injury and the overall patient’s status and hemodynamic stability.

**Figure 5:**
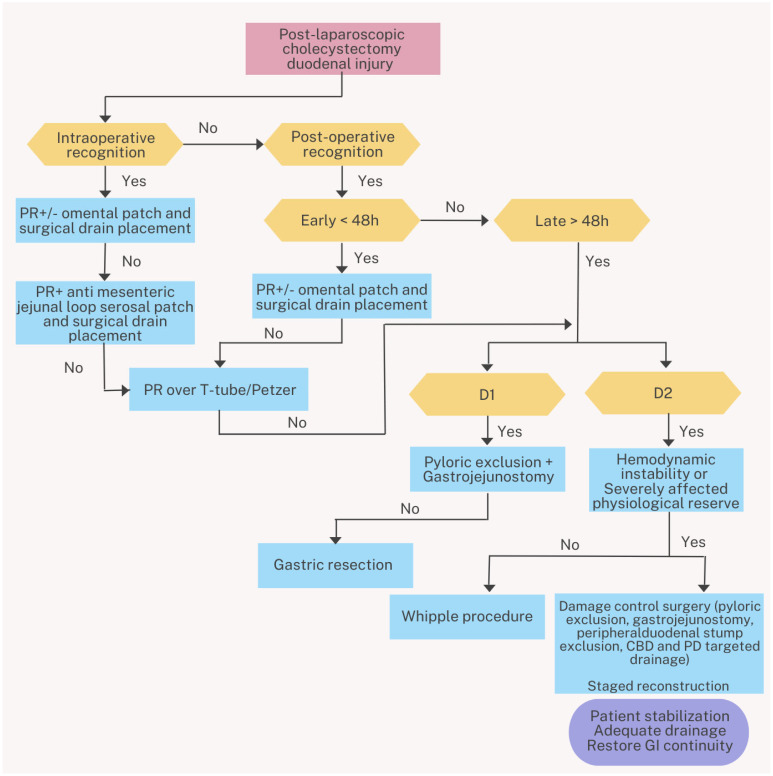
Algorithm of post-laparoscopic cholecystectomy duodenal injury surgical treatment. PR: Primary repair, D1: First portion of the duodenum, D2: Second portion of the duodenum, CBD: Common bile duct, PD: Pancreatic duct, GI: Gastrointestinal.

As in our case presentation, the treatment plan was personalized depending on the clinical parameters of our patient and was altered accordingly. There is no “one-size fits all” in terms of treatment plan. Timely diagnosis of the injury could present the opportunity of primary repair. In more extensive injuries this type of repair may not be feasible and more complex repairs could be required. The patient’s status should always be taken into consideration. In our case, when the plan seemed ineffective and the patient deteriorated, a damage-control approach was followed. Stabilization of the patient was our main concern. At first, staged-repair was considered appropriate. However, the spontaneous “neoduodenum” formation made further surgical repairs unnecessary. We present a treatment algorithm of Post-Laparoscopic Cholecystectomy Duodenal Injury in Figure 5.

## 5. Conclusions

LC is one of the most common operations performed worldwide. Complications following LC are rare, and most are related to bile duct injuries. Duodenal injuries are extremely rare but require vigilance and timely intervention. Various surgical approaches can be used depending on the time of injury recognition, location, and extent of the defect. In the presence of such injuries, surgeons must be vigilant for any postoperative complication by continuously evaluating the patient. According to the literature, immediate recognition, before patient’s clinical decompensation, has significant benefits, with an impact on both postoperative morbidity and mortality. Furthermore, upon identification of such complications, whether intraoperatively or postoperatively, the immediate transport of the patient to a specialized referral center should be the main priority. A personalized appropriate treatment of the patient from the beginning significantly increases the chances of achieving optimal outcomes.

## Figures and Tables

**Table 1:** Timeline of patient’s clinical course.

Day			Event	Procedure
Post-op after LC	Hospitalization at referral center	Post-op after damage control surgery		
6th	-	-	Bilious drain outflow Transfer to referral center	Nasogastric tube iv antibiotics TPN iv hydration iv somatostatin
7th	2nd	-	1st bleeding episode	Resuscitation CTA GDA elective embolization
11th	6th	-	2nd bleeding episode	Resuscitation CTA GDA embolization
14th	8th	-	3rd bleeding episode	Damage control surgery ICU
15th	9th	1st	-	Returning to ward
24th	18th	10th		Restoration of gastrointestinal function
26th	20th	12th	Bilious surgical drain outflow Null Kehr’s T-tube outflow	-
28th	22nd	14th	Fever (38.5 °C)	Wound drainage/debridement CT: intrabdominal abscess, pyloric recanalization Cholangiography: Kehr’s T-tube dislocation, stumps blowout
37th	31st	23rd	Fever-Fungemia	iv antibiotics modification
67th	61st	53rd	-	Discharge
3 months	1st of second hospitalization	-	Pre-renal acute renal injury	iv hydration CT: duodenal fistulous tract formation Surgical drain removal
	2nd	-	-	US control Discharge
1 year	-	-	-	Follow-up

iv: Intravenous, CTA: Computed tomographic angiography, GDA: Gastroduodenal artery, CT: Computed tomography, ICU: Intensive care unit, US: Ultrasound.

**Table 4:** Literature review on treatment procedures for post-laparoscopic cholecystectomy duodenal injuries.

Type of Procedure	Number of Patients	References	Death	Further Procedure Needed	Type
Surgical					
Primary Repair	42	[12,15,16,17,19,20,22,23,24,25,26,27,28,29,36,37,38,39,42,48,50], our	5 [12,17,18,19,22,38]	3 [18,42], our	NA [18] Endoscopic [42] Pyloric exclusion + Roux-en-Y gastrojejunostomy + biliary diversion + surgical drainage of pancreatic fluids + peripheral duodenal stump closure our
Omental patch	5	[16,17,36,42] , Our	1 [17]	2 [42], our	Our Endoscopic [42]
Over t-tube	3	[18,23]	0	0	
Tube duodenostomy	2	[23,40]	1 [23]	2 [23,40]	Gastric resection [23] PR (larger injury) + Tube duodenostomy (smaller injury) + Pyloric Exclusion [40]
Gastric resection	3	[30,43]	1 [30]	0	
Pyloric exclusion	1	[44]	0	0	
Omental patch	1	[45]	0	0	
Serosal patch	1	[30]	1 [30]	0	
Duodenopancreatectomy	1	[23]	0	0	
Roux-en-Y duodenojejunostomy	1	[23]	0	0	
**Conservative**	6	[16,30,36,41,46,47]	1 [30]	0	
**Endoscopic**	2	[31,49]	0	0	

NA: Not available, PR: Primary repair.

## Data Availability

No new data were created. The case presented was hospitalized in our hospital and our review was carried out using the existing literature. The review protocol can not be accessible. For any further information contact the authors.

## Supplementary Materials

The following supporting information can be downloaded at: https://www.mdpi.com/article/10.3390/jpm16030131/s1, Table S1: PRISMA 2020 Checklist.

## Abbreviations

The following abbreviations are used in this manuscript:

LC	Laparoscopic Cholecystectomy
FJ	Feeding Jejunostomy
WBC	White blood cells
CRP	c-Reactive Protein
TPN	Total Parenteral Nutrition
WSES	World Society of Emergency Surgery
AAST	American Association for the Surgery of Trauma
CT	Computed Tomography
GDA	Gastroduodenal artery
